# Exploring the Synergistic Association Between Oral Health Status and Oral Health Literacy Among College Students: A Cross-Sectional Study

**DOI:** 10.7759/cureus.41885

**Published:** 2023-07-14

**Authors:** Priya Agarahari, Ankita Jain, Souvir Mohan Pandey, Ajay Kumar Agrahari, Jagriti Yadav, Rangoli Srivastava, Sasmita Dalai, Tannu Kumari, Ashutosh K Singh, Yashi Sharma

**Affiliations:** 1 Department of Public Health Dentistry, Teerthanker Mahaveer Dental College and Research Centre, Moradabad, IND; 2 Department of Prosthodontics, Hazaribag College of Dental Sciences and Hospital, Hazaribag, IND; 3 Department of Anaesthesiology and Critical Care Medicine, Dr. Ram Manohar Lohia Institute of Medical Sciences, Lucknow, IND; 4 Department of Public Health Dentistry, Adesh Institute of Dental Sciences and Research, Bhatinda, IND

**Keywords:** reald, college students, oral health status, oral health literacy, health literacy

## Abstract

Introduction: Oral health is often viewed as a significant component for an indication of good general health or for good well-being together with a decent quality of life. Health literacy is considered a crucial factor in improvising a good life or excellent health. Oral health literacy (OHL) is the ability necessary for people to identify the factors that lead to poor oral health, learn and put into practice the essentials of effective oral self-care behaviors, and communicate with oral healthcare professionals in order to schedule appointments, put their names on waiting lists for dental care, and locate the dentist's office.

Aim and objectives: To evaluate students' oral health, their OHL, to determine the relationship between their oral health status and OHL, and finally to suggest preventive measures for the benefit of public health.

Materials and method: At Teerthanker Mahaveer University, a cross-sectional study involving 1500 participants, ages 18 to 25, was conducted on students studying nursing, physiotherapy, paramedicine, engineering, and law. Their informed consent was obtained. The Rapid Estimate of Adult Literacy in Dentistry (REALD-99) was used to gauge OHL levels, and the WHO's 1997 Oral Health Survey was used for their clinical evaluation.

Results: The mean REALD score was significantly higher in nursing students (88.32±6.46), followed by physiotherapy college (82.46±9.11), paramedical college (70.54±10.95), law college (46.52±7.74), and least in engineering college (38.80±10.65). The difference in the REALD score based on college was statistically significant. Along with this, the REALD score showed a correlation with gender and location too. Except for fluorosis, all the clinical parameters of dental caries, gingival bleeding, and pockets, loss of attachment, dental fluorosis, and dental enamel were associated with OHL.

Conclusion: The results of the current study showed a relationship between educational attainment, clinical parameters examined, and OHL, leading to the conclusion that higher OHL is related to better oral health. So, we can conclude that maintaining good oral health requires OHL.

## Introduction

One of the fundamental factors that have been taken into account with regard to one's health is the improvement of one's capacity to maintain health, recover from illness, and enhance QOL (quality of life). Knowing how to read health education booklets, prescription instructions, doctor's instructions, and consent forms is an indicator of health literacy [1]. Despite the fact that healthcare professionals typically presume that patients and their families are given clear health explanations and instructions, in practice these directions are frequently misunderstood, which might result in errors. Lack of health literacy in patients may be a typical cause of misinterpretation of medical recommendations [2,3].

Professor Scott K. Simonds coined the phrase "health literacy" in his 1974 work, Health Education as Social Policy. The World Health Organization defined health literacy as "cognitive and social abilities that influence an individual's ability to obtain, comprehend, and apply knowledge in ways that preserve oral health" [4].

Although the majority of patients get their information from a variety of sources, the advice given by their dentist and the dental team members serves as a guide for the best oral health-related decisions in relation to general health [5]. During their visit to the dentist, patients have the opportunity to get advice and access extra medical services. The dental team's communication abilities help patients become more knowledgeable about their oral health, which leads to better oral health outcomes [6].

All the necessary skills are required so that a person is aware of all the factors relating to a dental practice, such as when to visit a dental clinic, how to keep your mouth clean, how to schedule a dental appointment, and how to travel there. All such information can be taken into consideration for oral health literacy (OHL) [7].

People with lower levels of health literacy are more likely to have poor health understanding and status behavior, lower rates of using preventive services, higher hospitalization rates, higher costs of medical care, and inevitably experience worse health results than people with higher literacy rates [8]. It has been shown that socioeconomic factors like race, education, health behaviors, and health outcomes can all be mediated by health literacy, which can help explain some elements of health disparities [9].

Both the public's understanding of the importance of literacy in dentistry and the efforts that have been made to apply the concept of health literacy to dental research and practice have progressed over time [10]. Because there are not enough methods for evaluating OHL at this time, it is not possible to investigate the evidence relating to the individual's poor dental condition and how it had been brought about by an inadequate understanding of oral health.

Therefore, the current study was conducted with the goal of assessing the association between OHL and oral health status among Teerthanker Mahaveer University students aged 18 to 25.

## Materials and methods

A cross-sectional study was conducted to assess OHL levels and correlate the impact on oral health status among students of Teerthanker Mahaveer University in Moradabad district, India from May 2020 to April 2021. Students were assessed for their clinical examination and the questionnaire too, and in total, we took 300 students from each of them. Based on the pilot study we conducted and the results obtained, we made an estimate for the final study’s sample size by simple random sampling, which came out to be 1500 (males: 864, females: 636). For the present study, the sample size was determined using the formula, n=(Z^2*p*(1-p))/(E^2), where n is the required sample size, Z is the z-score corresponding to the desired level of confidence (e.g., for a 95% confidence level, Z would be 1.96), p is the estimated proportion or prevalence of the characters in the population, E is the desired margin of error or precision (expressed as a proportion) at a 95% confidence interval. The students were taken purposefully from those colleges that did not have any dental backgrounds.

Ethical clearance was obtained from the Teerthanker Mahaveer Dental College with institutional review board number: TMDCRC/IEC/20-21/PHD5. Permission from the respective colleges and authorities was obtained, and informed consent was obtained from all the participants prior to the study. Inclusion criteria include students aged between 18 and 25, students enrolled in nursing, physiotherapy, paramedical, engineering, and law disciplines at Teerthanker Mahaveer University, and students who were willing to participate in the research. Exclusion criteria include those who refused to provide informed consent, suffered from any systemic diseases or were undergoing orthodontic treatment.

Data collection

The principal investigator obtained data from respondents through an oral examination using the Rapid Estimate of Adult Literacy in Dentistry (REALD-99) instrument and a modified WHO proforma (1997) [11,12]. The questionnaire was in English and translated to the local language; its validation was done by experts in the field where the face value was 1 and Cronbach's alpha value was 0.8. OHL was evaluated using the REALD-99. For this previously investigated word recognition test, strong psychometric characteristics have been found. It has 99 dental-related statements, arranged in descending complexity. The participants were instructed to read the words aloud. They were told to read only words that they understood and could pronounce clearly. Each word that is properly uttered by the participants receives one point toward the REALD-99 score. These points are summed up to determine the final score. The range of the score is 0 (low literacy) to 99 (high literacy). The WHO Oral Health Survey Adult Oral Health Questionnaire, 5th edition, was used to measure oral health-related behavior. The questionnaire was adjusted and pretested in accordance with the study's requirements. The components of the WHO Oral Health Survey were recorded, and the oral examination was done under natural light with the participants sitting in a chair. The investigator was accompanied by a professional recorder who assisted in the data collection. 

Statistical analysis

The data for the present study was entered and analyzed using the Statistical Package for the Social Sciences (SPSS 19.0 Version). The descriptive statistics included the mean and standard deviation. The intragroup comparison for the different time intervals was done using a paired t-test and the correlation was established based on the answers. Shapiro-Wilk test was used for normality, where a non-significant p-value (p>0.05) suggested that the data were normally distributed. The level of significance for the present study was fixed at 5%. The intergroup comparison for the difference in mean scores between independent groups was done using the unpaired/independent t-test and one-way ANOVA.

## Results

**Figure 1:**
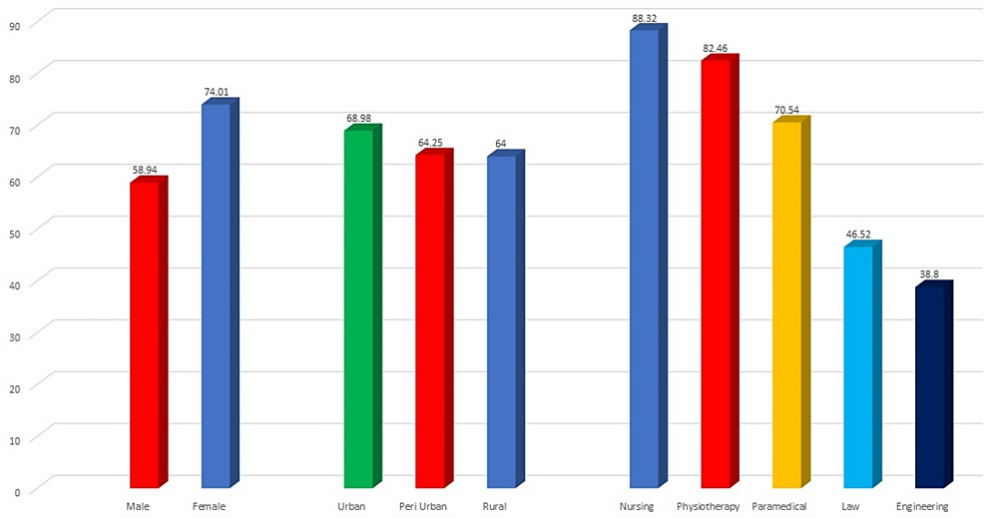
Mean REALD scores comparison

Among the study subjects, 42.4% of the subjects were females and 57.4% were males. The mean REALD score among the females was 74.01±19.17 and among the males was 58.94±20.99. The intergroup comparison of the mean REALD score was statistically significant between the males and females. Based on the location, 24% of the subjects were from rural areas, 52.8% were from peri-urban areas, and 23.20% were from urban areas. The mean REALD score was highest in urban areas (68.98±23.1) followed by periurban areas (64.25±20.24) and least in rural areas (64.00±22.39). The difference in the REALD score based on location was statistically significant. Based on the college, 20% were from nursing college, physiotherapy college, paramedical college, law college, and engineering college. The mean REALD score was significantly higher in nursing college (88.32±6.46) followed by physiotherapy college (82.46±9.11) and least in engineering college (38.80±10.65). The difference in the REALD score based on college was statistically significant when analyzed using one-way ANOVA (Table 1, Figure 1).

**Table 1:** Distribution of subjects by gender and its association with REALD scores REALD, Rapid Estimate of Adult Literacy in Dentistry; SD, standard deviation

	Number of Subjects (n)	Mean REALD Score ± SD	P-Value
GENDER			0.001
Male	864	58.94±20.99	
Female	636	74.01±19.17	
LOCATION			0.001
Urban	348	68.98±23.1	
Peri Urban	792	64.25±20.24	
Rural	360	64.00±22.39	
COLLEGES			0.001
Nursing	300	88.32±6.46	
Physiotherapy	300	82.46±9.11	
Paramedical	300	70.54±10.95	
Law	300	46.52±7.74	
Engineering	300	38.80±10.65	

Among the study subjects, 917 subjects had sound teeth, 430 subjects had caries, 20 subjects had filled teeth with caries, 43 subjects had filled teeth with no caries, 15 subjects had missing teeth due to other reasons, and 75 subjects had missing teeth due to caries. The difference in the REALD score based on dentition status was statistically significant when analyzed using an independent t-test. Caries were seen in 583 subjects. The mean REALD score was higher in the caries-free subjects (70.02±21.69) compared to subjects with caries (60.23±21.34). The difference in the REALD score based on caries status was statistically significant when analyzed using independent t-test. Gingival bleeding was seen in 678 participants. The mean REALD score was higher in the subjects without gingival bleeding (67.06±21.89) compared to subjects with gingival bleeding (63.22±20.98). The difference in the REALD score based on gingival bleeding was statistically significant when analyzed using independent t-test. 

Gingival pockets were seen in 336 subjects. The mean REALD score was higher in the subjects without shallow pockets (69.33±21.58) compared to subjects with shallow pockets (61.30±21.52). The difference in the REALD score based on shallow pockets was statistically significant when analyzed using independent t-test. Among the study subjects, 1326 subjects were having no loss of attachment (LOA), 120 subjects were having LOA score 2, and 36 patients were having LOA Score 1. The mean REALD score was higher in the subjects without LOA (88.00±0.01) compared to subjects with LOA. The difference in the REALD score based on LOA was statistically significant.

**Figure 2:**
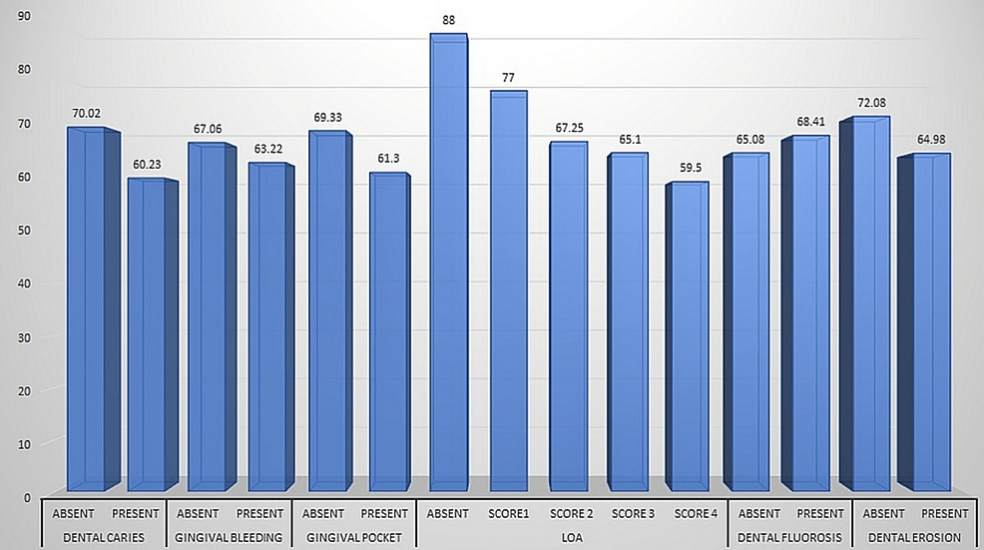
Distribution of study subjects based on dichotomized outcome variables and their associations with REALD scores

Dental fluorosis was seen in 72 subjects. The mean REALD score was lower in the subjects without dental fluorosis (65.08±21.61). The difference in the REALD score was statistically non-significant (p-value=0.021). Dental erosion was seen in only 72 participants. The mean REALD score was higher in the subjects without dental erosion (72.08±18.68). The difference in the REALD score was statistically significant (p-value=0.001) (Table 2, Figure 2).

**Table 2:** Distribution of study subjects based on dichotomized outcome variables and their associations with REALD scores REALD: Rapid Estimate of Adult Literacy in Dentistry

Clinical Parameters	Number of Subjects (n)	Mean REALD Score (±SD)	P-value
Dental Caries			0.001
Absent	917	70.02±21.69	
Present	513	60.23±21.34	
Gingival Bleeding			0.001
Absent	822	67.06±21.89	
Present	678	63.22±20.98	
Gingival Pocket			0.001
Absent	1164	69.33±21.58	
Present	336	61.30±21.52	
LOA			0.001
Absent	1326	88.00±0.01	
Score1	36	77.00±1.04	
Score 2	120	67.25±23.95	
Score 3	06	65.10±21.48	
Score 4	12	59.50±17.63	
Dental Fluorosis			0.021
Absent	1422	65.08±21.61	
Present	72	68.41±20.69	
Dental Erosion			0.001
Absent	1428	72.08±18.68	
Present	72	64.98±21.64	

## Discussion

Oral health is a significant predictor of overall health, happiness, and QOL. Health literacy is increasingly being referred to as a currency for enhancing health and healthcare quality. A person's ability to function in the healthcare system is dependent on fundamental reading and numeracy skills. It is assumed that OHL depends on socioeconomic factors and the lifestyle of a person, and there have been many studies conducted to assess its relationship with other demographic factors. Hence, this present study was conducted among college students from different colleges at Teerthanker Mahaveer University.

In the study conducted by Sistani et al., they assessed 1031 adults' oral health behavior (OHB) by noticing their toothbrushing frequency and direction pattern and further correlated it with OHL with the help of a validated questionnaire, concluding that OHL was related to OHB, while in our study, we took 1500 adults and assessed their clinical status and further correlated it with a significant association with all clinical factors except enamel fluorosis [13]. In the study conducted by Wong HM et al., they developed and validated a new REALD scale to assess literacy in the dental field, while in our study we used an already evaluated version of REALD [11]. We also validated this scale in our pilot study [14].

In a study by Haridas et al., they evaluated OHL using the REALD-30, evaluated occupational health and safety (OHS) among adults attending dental college, and clinically evaluated decayed, missing, filled teeth (DMFT), community periodontal index (CPI), and LOA [15]. Only DMFT was negatively associated with the REALD score, while in our research, OHL was assessed with the help of the REALD-99, and OHS was assessed among different college students. Clinically assessing DMFT, CPI, and LOA showed a positive correlation. In a study conducted by Sandhu et al. on health literacy and OHL correlation with the help of a rapid estimate of adult literacy in medicine (REALM) and REALD, respectively, concluded that the relationship between health literacy and OHL was not strong; whereas, in our research, we assessed the OHL and oral health status with the help of the REALD instrument and OHS by WHO-modified proforma (2013) and concluded that the association between OHL and OHS was statistically significant [16].

Wimardhani et al. assessed 1000 adults from five different areas of Jakarta and evaluated them using the health literacy in dentistry (HeLD-ID) score, which revealed a significant relationship with all sociodemographic details except gender [17]. In contrast, in our study, we correlated OHL with the clinical status of 1500 college students and discovered a positive correlation with OHL except for one clinical factor, namely enamel fluorosis.

In the study conducted by Simon et al., after assessing the OHS of 260 school teachers and further correlating it with OHL, the authors concluded that OHL was significantly related to filled teeth and level of education, while in our study, we found that not only filled teeth but decayed and missing teeth, along with the level of education, were positively related to OHL [18]. In the study conducted by Batista et al., participants were clinically assessed and sociodemographic data were recorded. OHL was assessed with the help of a Likert scale, and it showed a significant association with sociodemographic details, while in our study, we took college students and noted their clinical as well as educational details, both of which were positively associated with OHL [19].

With the aid of the REALD-30 score, Kesavan et al. evaluated OHL among students at a private university in Chennai [20]. It was seen to be low among those students who belong to lower socioeconomic status and vice versa, while in our study, those students who belong to rural areas had a lower OHL compared to urban area college students. A study conducted by Jaafar A et al. assessed the OHL of university UG students with the help of the Malaysian version of the validated OHL index, and it showed that females had a higher OHL compared to males, while in our study, gender was also discovered to have a substantial relationship with OHL [21].

The study's limitations were that it was cross-sectional in design and did not investigate the cause-and-effect relationship. Because reading is regarded as intermediate to decoding and comprehension, we were only able to assess a person's reading skills using REALD instrument. English fluency was also a limiting factor in this study since several individuals were unable to adequately pronounce words while having an understanding of them. The results of this particular cross-sectional study can not be generalized, as we only took a section of the population of adults belonging to the age group of 18-25 years.

## Conclusions

The strongest correlation between REALD 99 and oral health status was found in nursing followed by physiotherapy, paramedicine, law, and engineering. As a result, we can say that there was a correlation between OHL and oral health state changes with the stream. The following oral health outcomes had strong correlations with OHL: DMFT, CPI, LOA, enamel erosion, and dental trauma. The lowest OHL that was unrelated to OHL is enamel fluorosis. The burden of a lower literacy level among adults who have a significantly lower OHL should be lessened, and as public health dentists, it should be our duty to do so. The clinical factors that cause the oral health of this group to deteriorate and further lower their OHL score should be monitored, diminished, and prevented.
